# Characteristics of mast cell infiltration in lung adenocarcinoma and its impact on prognosis

**DOI:** 10.1007/s12672-024-01062-5

**Published:** 2024-06-04

**Authors:** Zijian Qiu, Guanchao Pang, Xia Xu, Jun Lin, Pingli Wang

**Affiliations:** 1 Department of Radiation Oncology, The Quzhou Affiliated Hospital of Wenzhou Medical University, Quzhou People’s Hospital, Quzhou, Zhejiang China; 2 Department of Respiratory and Critical Care Medicine, The Second Affiliated Hospital of Zhejiang University School of Medicine, No. 1511, Jianghong Road, Hangzhou, 310003 China; 3 Department of Pathology, The Second Affiliated Hospital of Zhejiang University School of Medicine, Hangzhou, Zhejiang China; 4 Department of Pathology, The Quzhou Affiliated Hospital of Wenzhou Medical University, Quzhou People’s Hospital, Quzhou, Zhejiang China

**Keywords:** Lung adenocarcinoma, Mast cells, Microvessels, Prognosis

## Abstract

**Background:**

The role of mast cells in malignancies remains unclear, and there is no clear correlation between mast cells and tumor microvessels, tumor growth, or lung adenocarcinoma (LUAD) prognosis. This study aims to explore the association between mast cell density (MCD) and intratumoral microvessel density (MVD), clinicopathological parameters, and prognosis in LUAD, by evaluating mast cell infiltration characteristics and their prognostic significance.

**Methods:**

This retrospective investigation involved 238 patients with LUAD undergoing complete resection. Tumor and normal lung tissue sections outside the tumor were immunohistochemically stained for MCD in the intratumoral and outside regions, respectively. CD34 polyclonal antibody was used to measure intratumoral MVD.

**Results:**

Intratumoral regions of LUAD had a higher MCD (*P* < 0.001) than normal lung tissue. In the intratumoral region, MCD and CD34-MVD were positively correlated (r = 0.411, *P* < 0.001). Intratumoral MCD correlated with sex, smoking history, tumor differentiation, pathological subtype, and tumor size. Female sex (*P* = 0.012), no smoking history (*P* = 0.002), acinar predominant type (*P* = 0.012), and tumor size ≤ 3 cm (*P* = 0.009) were associated with a higher MCD, whereas poorly differentiated (*P* = 0.039) and solid/micropapillary predominant types (*P* = 0.001) were associated with a lower MCD. Higher intratumoral MCD exhibited a marginally improved overall survival, and individuals with higher MCD infiltration ratios (intratumoral MCD/outside the MCD) had higher disease-free and overall survival rates (log-rank *P* < 0.001). A high MCD infiltration ratio was associated with decreased risk of tumor progression and death following complete resection.

**Conclusion:**

The tumor microenvironment controls mast cell infiltration in LUAD, and patients with increased intratumoral mast cell infiltration have better prognosis.

## Introduction

Lung cancer is a type of malignant tumor that has the highest rate of morbidity and mortality worldwide [1]. Non-small cell lung cancer (NSCLC) accounts for approximately 85% of cases of lung cancer [2]. Lung adenocarcinoma (LUAD) is the most common type of NCSLC and is the main pathological type responsible for lung cancer-related morbidity and mortality. Surgical resection is the best treatment for patients with early-stage LUAD. The longevity in patients with LUAD has improved, evidenced by the declining death rate in recent years. However, despite complete resection, many patients still have significant risks of death and recurrence [3].

Currently, TNM staging is useful for assessing patient prognosis and indicating the extent of tumor progression [4]. Additionally, the prognosis in patients is significantly affected by the various pathological subtypes of LUAD [5, 6]. Tumor stage and histological classification are used to determine the prognosis in patients with LUAD. Tumor growth, metastasis, and treatment are closely correlated with the number of stromal cells in the tumor microenvironment, including tumor-associated fibroblasts, immune cells, and endothelial cells [7], in particular, the considerable abundance of tumor-infiltrating immune cells [8]. Thus, investigating the efficient and targeted immune cells that infiltrate tumors is clinically valuable for assessing the prognosis of LUAD.

Although mast cells are vital components of the immune cell family, they are also involved in the onset and progression of malignancies [9]. An increased number of mast cells in lung cancer has been linked to tumor growth and poor patient prognosis [10–13]. Although the number of mast cells in the intratumoral zone is not correlated with the prognosis of NSCLC patients [14, 15], patients with a high number of mast cells in the peritumoral area have a lower probability of death by stage I NSCLC [14]. However, other studies have reported that a higher quantity of mast cells in NSCLC tumor tissues improves patient prognosis [16, 17]. Therefore, this disagreement between studies underscores the complex association between mast cells and NSCLC prognosis. Recently, mast cells have been shown to create angiogenic and lymphangiogenic chemicals, which may play a role in inflammation and tumor angiogenesis [18]. However, extensive research on this subject and conflicting findings regarding the association between mast cells and angiogenesis in NSCLC are lacking [19].

Both mast cell chymase and tryptase enzymes are considered as prominent markers of mast cells [20]. The classification of mast cells is based on the specific enzymes they possess: mast cells with tryptase exclusively, mast cells with both chymase and tryptase, and those with chymase exclusively [21]. Mast cells contain the highest concentration of tryptase [22], and their chymase content could be 10 times lower than their tryptase levels [23].

Only a few researchers have employed anti-chymase antibodies in their studies on mast cells and lung cancer. For example, Carlini et al. [14] and Shikotra et al. [17] reported that of 65 and 49 patients with NSCLC, those with LUAD were even fewer; neither of these studies included patients with LUAD examined in isolation. Nagata et al. [12] included patients only with small (tumor diameter ≤ 2.0 cm) LUAD. No studies have used anti-mast cell chymase antibody to stain LUAD specimens isolated from patients following full resection of stage I–III disease. Consequently, it is currently unclear how many chymase-labeled mast cells infiltrate patient tumor tissues after LUAD surgery, and what is the relationship between tumor microvessels, tumor development, and patient prognosis.

In this study, the anti-mast cell chymase antibody CC1 was utilized as a mast cell marker to evaluate mast cell density (MCD), and CD34 as a pan-vascular endothelial cell marker was used to analyze and measure microvessel density (MVD). Our findings may be useful for determining the features of mast cell infiltration and their prognostic value in LUAD.

## Methods

### Study subjects and enrollment conditions

We utilized data of patients with LUAD who underwent full resection at the Second Affiliated Hospital, Zhejiang University School of Medicine, between January 1, 2011, and December 30, 2015. Requirements for case admission eligibility were as follows: (1) a clear pathological diagnosis of LUAD and no history of other cancers post surgery; (2) stage I–III; (3) chemotherapy and radiation were not used as preoperative adjuvant therapy in any of the patients; (4) no history of allergic reactions, including asthma; and (5) the patient’s lung cancer tissue samples were formally collected by the pathology department and the tumor tissue was sectioned in paraffin blocks to produce tissue sections that were well preserved and had a thickness of 3–5 μm. Patients with distant metastases were excluded from the study.

A total of 238 patients (133 female and 105 male) aged 26–87 years (mean age, 60.48 years) were enrolled. Table 1 provides further information on clinicopathological features.

**Table 1:** Connection between clinicopathological features and MCD in the intratumoral area

Feature grouping	Number	Proportion (%)	$\overline{x }$±s	Difference value	95% CI	t-test/F test	
						T/F Value	*P* Value
Sex							
Female	133	55.9	14.37 ± 7.966	2.435	0.543–4.327	2.536	0.012
Male	105	44.1	11.93 ± 6.835				
Tumor site							
Left Lung	106	44.5	13.04 ± 7.801	−0.462	− 2.411–1.486	− 0.467	0.641
Right Lung	132	55.5	13.50 ± 7.404				
Age, years							
≤ 60	108	45.4	12.93 ± 7.246	−0.674	− 2.618–1.270	−0.683	0.495
> 60	130	54.6	13.60 ± 7.845				
Smoking history							
Never-smoker	166	69.7	14.22 ± 7.928	3.070	1.182–4.958	3.210	0.002
Smokers	72	30.3	11.15 ± 6.211				
Tumor differentiation							
Well	59	24.8	13.81 ± 8.887	/	11.50–16.13	2.932	0.039
Moderately	85	35.7	14.51 ± 7.320		12.93–16.08		
Poorly	94	39.5	11.87 ± 6.697		10.50–13.24		
Pathological subtypes							
Acinar	127	53.4	14.45 ± 7.795	/	13.08–15.82	2.853	0.016
Papillary	31	13.0	12.29 ± 6.905		9.76–14.82		
Micropapillary	9	3.8	11.67 ± 6.481		6.69–16.65		
Lepidic	35	14.7	14.29 ± 8.774		11.27–17.30		
Solid	18	7.6	8.83 ± 3.930		6.88–10.79		
Variant	18	7.6	10.22 ± 5.397		7.54–12.91		
Pathological subtypes							
Solid/Micropapillary	27	11.3	9.78 ± 4.987	− 3.966	− 6.178−1.754	− 3.614	0.001
Others	211	88.7	13.74 ± 7.734				
Pathological subtypes							
Acinar	127	53.4	14.45 ± 7.795	2.476	0.560–4.392	2.546	0.012
Non-Acinar	111	46.6	11.97 ± 7.113				
Tumor Size							
≤ 3 cm	131	55.0	14.44 ± 7.753	2.555	0.635–4.475	2.622	0.009
> 3 cm	107	45.0	11.89 ± 7.127				
T Stage							
T1	68	28.6	13.91 ± 6.646	/	12.30–15.52	0.300	0.792
T2	118	49.6	12.86 ± 7.163		11.55–14.16		
T3	32	13.4	13.34 ± 8.983		10.11–16.58		
T4	20	8.4	13.70 ± 10.433		8.82–18.58		
N Stage							
N0	132	55.5	13.80 ± 8.112	1.126	− 0.782–3.033	1.162	0.246
N +	106	44.5	12.67 ± 6.824				
TNM stage							
Stage I	74	31.1	13.80 ± 7.266	/	12.11–15.48	1.090	0.338
Stage II	73	30.7	12.21 ± 7.846		10.37–14.04		
Stage III	91	38.2	13.76 ± 7.581		12.18–15.34		

CI: confidence interval; MCD: mast cell density

### Obtaining clinicopathological data

Through the hospital information system, all patient clinicopathological data, including name, age, gender, smoking history, tumor location, size, degree of differentiation, pathological subtype, lymph node status, and TNM staging, were meticulously documented. A combination of phone consultations and hospital information system gathering was used to carry out the follow-up. Tumor progression (recurrence or metastasis), progression period, survival status, and time of death were also monitored. Disease-free survival (DFS) was calculated as the interval between the start of the resection procedure and tumor progression. Overall survival (OS) was calculated as the interval between LUAD diagnosis and death. Lung cancer staging was performed according to The Union for International Cancer Control (UICC), standard version 8. This study was approved by the Ethics Committee of the Second Affiliated Hospital of the Zhejiang University School of Medicine (Document batch number 2019-308). Informed consent was waived by our Ethics Committee because of the retrospective nature of our study. We confirm that all methods were performed in accordance with the relevant guidelines and regulations.

### Immunohistochemical staining

Prior to antigen repair, paraffin slices were deparaffinized in xylene and 100%, 85%, and 75% ethanol. Citric acid antigen repair solution was then applied. The sections were additionally exposed to endogenous peroxidase blocking using a 3% hydrogen peroxide solution. Subsequently, the histochemical ring was blocked with 3% bovine serum albumin for 30 min at room temperature. The sections were incubated for 1 h at room temperature following the addition of the primary antibodies. Antibodies used were anti-mast cell chymase antibody CC1 (Abcam, ab2377, Cambridge, United Kingdom) prepared at a ratio of 1:200 with phosphate-buffered saline (PBS) and polyclonal antibody CD34 (Proteintech, 14486-1-ap, Wuhan, China) prepared at a ratio of 1:800 with PBS. Hypersensitive mouse and rabbit universal secondary antibodies (RecordBio, RC0080RM, Shanghai, China) were applied to the tissues and incubated for 30 min at room temperature. Freshly synthesized diaminobenzidine (diluted and concentrated solutions prepared at a ratio of 1000:50) was added to create the hue. The color development period was monitored under a microscope, and the resultant positive color was brown-yellow. The nuclei were counterstained with hematoxylin. The final sections were then dehydrated and sealed with neutral gum. A digital pathological slice scanner (KFBIO, KF-PRO-120, Ningbo, China) was used to obtain slice images for microscopic examination.

### Interpretation of results

K-Viewer V1 (KFBIO, code 1.5.5.10, Ningbo, China) was used to read the slides. On hematoxylin staining, blue indicated the nucleus, and brown-yellow indicated the positive expression of diamine benzidine.

### Calculating the MCD and MVD

The Weider correction method [24] was applied, and three “hot spots” containing the highest number of microvessels and mast cells were identified for each specimen at low magnification (× 10). The number of microvessels and mast cells in each location were counted at high magnification (× 40), and the average value was determined using the MVD and MCD values.

### Statistic analysis

The statistical package SPSS 25.0 (IBM corporation, New York, USA) and R version 4.2.1 were used for data analysis. Rank correlation analysis was conducted to determine the relationship between MCD and MVD. A paired design t-test was used to compare the difference in MCD between intratumoral and normal areas. A t-test or F-test was used to analyze the correlation between MCD and clinicopathological characteristics. The Kaplan–Meier method was used to calculate survival rates, and the log-rank test was used to compare survival differences. Univariate and multivariate analyses were performed with the Cox hazards regression model to assess the prognostic factors. A *P* value of less than 0.05 was considered statistically significant.

## Results

### Infiltration of mast cells in LUAD

**Fig. 1:**
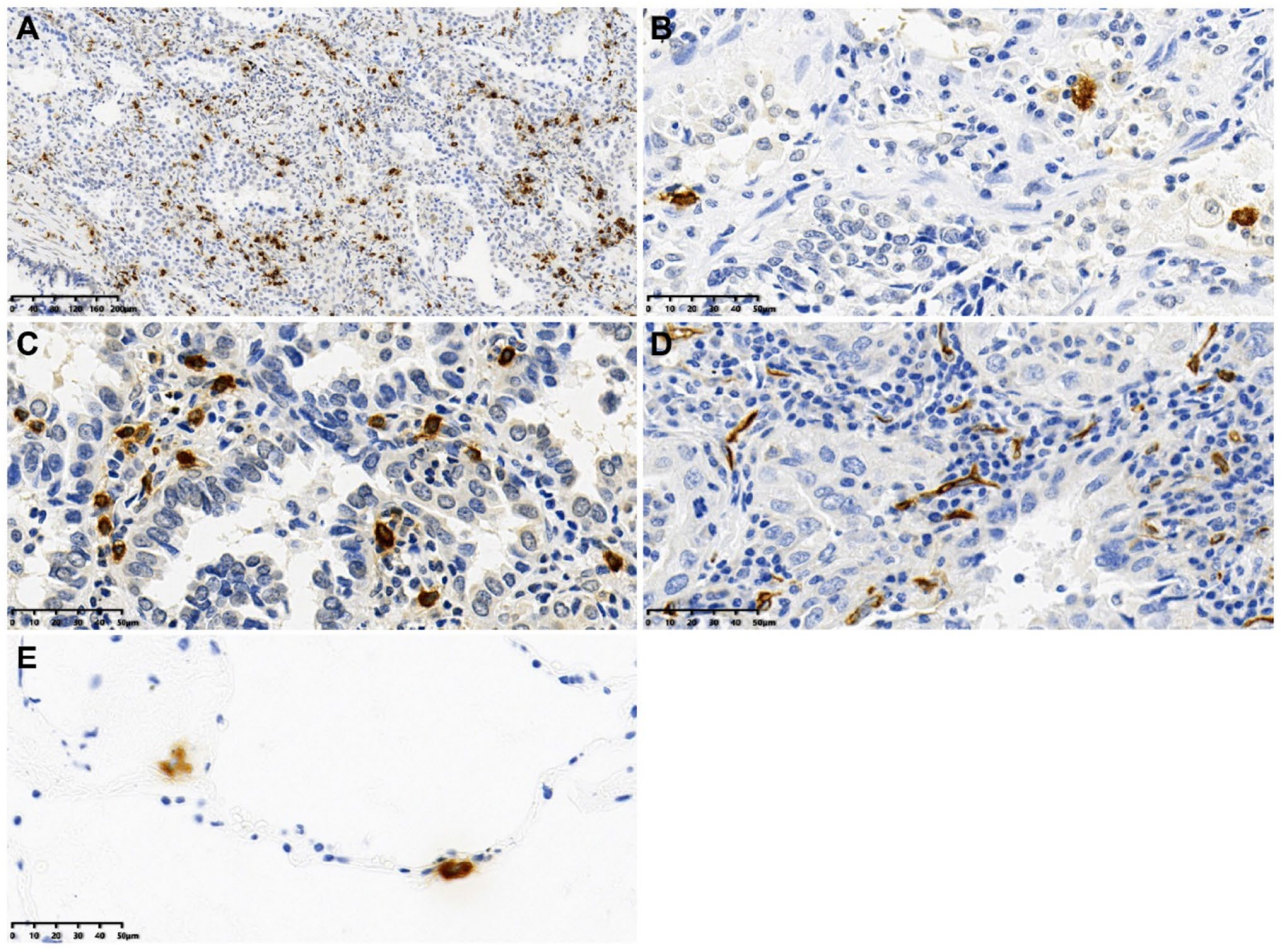
Expression of chymase-positive mast cells and CD34 microvessels in LUAD tissues. **A** The intratumoral mast cells are detected at low magnification of × 10. **B** and **C** The intratumoral mast cells are detected at high magnification of × 40, **B** shows a Low MCD group and **C** a High MCD group. **D** The intratumoral CD34-microvessels are detected at a high magnification of × 40. **E** The mast cells are detected in the normal lung tissue outside the tumor at high magnification of × 40. LUAD: lung adenocarcinoma; MCD: mast cell density; MVD: microvessel density

**Fig. 2:**
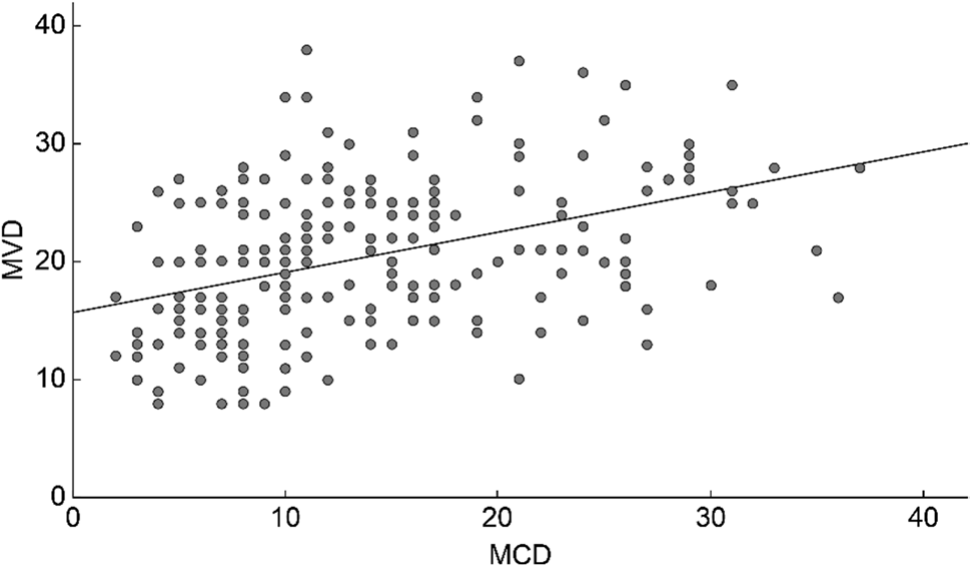
Scatter plot of intratumoral MCD and MVD. MCD: mast cell density; MVD: microvessel density

Both mast cells and CD34 microvessels were detected in LUAD (Fig. 1). In the intratumoral area, MCD ranged from 2 to 37 per high magnification (× 40) field of view, with an average of 13.29 ± 7.571. The MVD ranged from 8 to 38 per high magnification (× 40) field of view, with an average of 20.29 ± 6.352. A significant positive association was observed between MCD and MVD (Pearson's correlation coefficient r = 0.411, P < 0.001) (Fig. 2).

In the normal lung tissue outside the tumor, the MCD ranged from 1 to 6 per high-magnification (× 40) field of view, with an average of 2.49 ± 1.046. The MCD in the intratumoral area was significantly higher than that in the normal areas (difference value 10.807, 95%CI, 9.910–11.703, *P* < 0.001). The median infiltration ratio was 5.37 (4.00), which was determined by dividing intratumoral MCD by outside MCD.

### Relevance of MCD to clinicopathological features

Age, sex, tumor location, smoking history, pathological subtype, degree of differentiation, tumor size, tumor stage, and other clinicopathological features were not associated with MCD in normal lung tissue that was outside the tumor (*P* ≥ 0.05).

Intratumoral MCD was higher in female patients (T = 2.536, *P* = 0.012), non-smokers (T = 3.210, *P* = 0.002), those with acinar predominant type (T = 2.546, *P* = 0.012), and those with tumor size ≤ 3 cm (T = 2.622, *P* = 0.009), but lower in those with solid/micropapillary predominant type (T = − 3.614, *P* = 0.001) and poorly differentiated tumors (F = 2.932, *P* = 0.039). Age, tumor site, and TNM stage did not significantly alter MCD (*P* < 0.05) (Table 1).

### Survival analysis

**Fig. 3:**
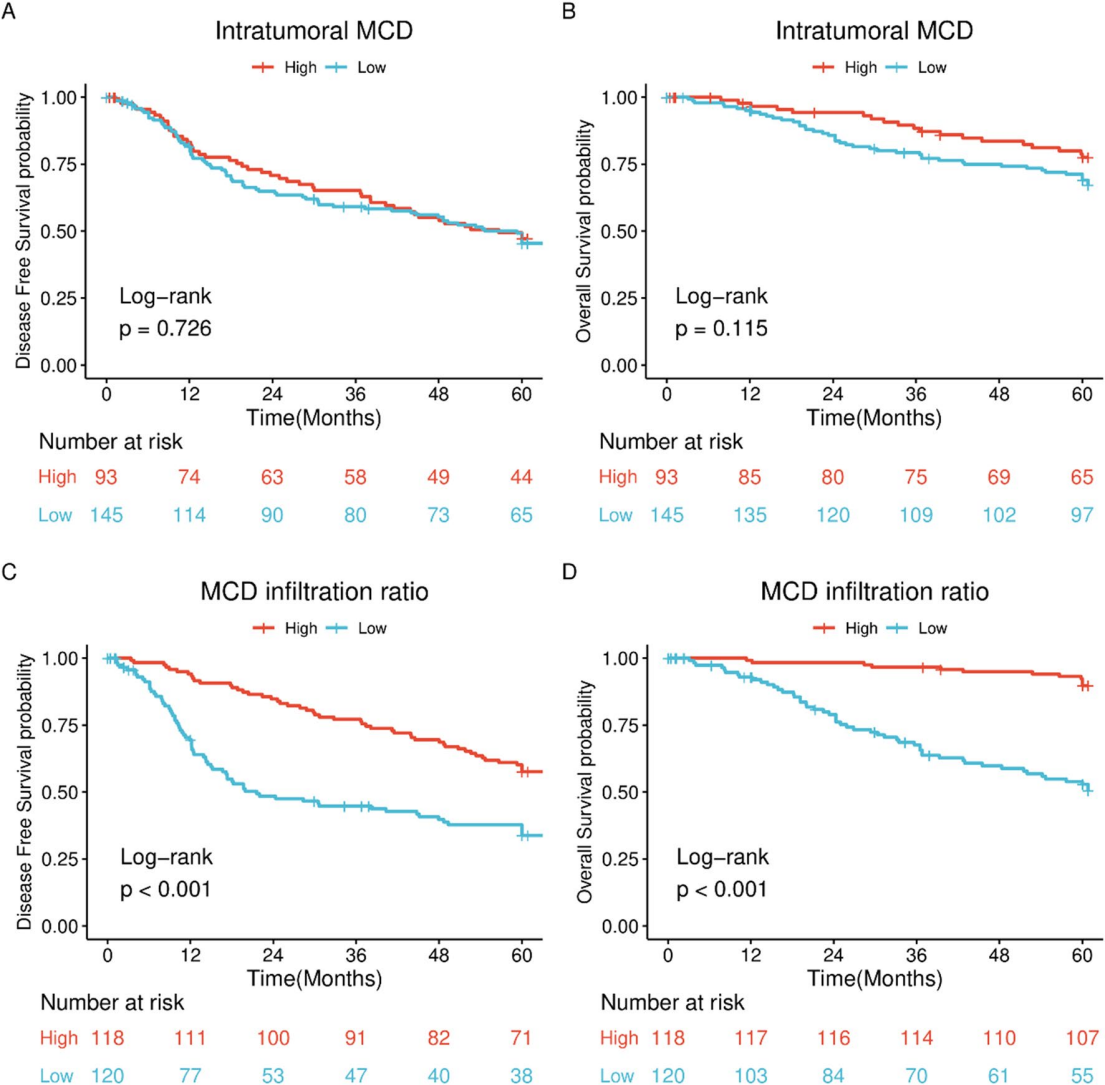
Survival analysis. **A** DFS curves in patients with different intratumoral MCD levels (Log rank P = 0.726). **B** OS curves in patients with different intratumoral MCD levels (Log rank P = 0.115). **C** DFS curves in patients with different MCD infiltration ratios (Log rank P < 0.001). **D** OS curves in patients with different MCD infiltration ratios (Log rank P < 0.001). DFS, disease-free survival; MCD: mast cell density; OS, overall survival

The mean value of intratumoral MCD was used as the boundary, we classified those with MCD ≤ 13 into the low MCD group and those with MCD > 13 into the high MCD group. Patients in the low MCD group had a 5-year DFS rate of 45.5%, whereas those in the high MCD group had a rate of 47.2%. Figure 3A shows no significant difference in DFS between the two groups (log-rank *P* = 0.726).

The low- and high-MCD groups had 5-year OS rates of 69.1% and 77.5%, respectively. Patients in the high MCD group had a higher OS rate than those in the low MCD group (Fig. 3B), although the difference between the two groups was not statistically significant (log-rank *P* = 0.115).

The median infiltration ratio served as the boundary for grouping the patients into high- and low-infiltration categories. Patients in the high infiltration group had higher disease-free and overall survival rates. Figure 3C and D show significant differences between the two groups (log-rank *P* < 0.001).

### Univariate and multivariate analysis of DFS and OS

We ran a univariate analysis of DFS and OS for every variable related to clinicopathological characteristics. Survival was correlated with tumor differentiation, TNM stage, lymph node metastasis, MCD invasion rate, and disease progression (*P* < 0.05). The above variables were included to construct a multivariate Cox proportional hazards model was constructed for disease progression and death in patients with LUAD following complete resection. A high infiltration ratio of MCD decreased the risk of tumor progression and death in patients with LUAD after complete resection (*P* < 0.001). The details are shown in Table 2.

**Table 2:** Univariate and multivariate analysis of DFS and OS

VARIABLES	DFS				OS			
	Univariate		Multivariate		Univariate		Multivariate	
	HR (95% CI)	*P* Value	HR (95% CI)	*P* Value	HR (95% CI)	*P* Value	HR (95% CI)	*P* Value
Sex								
Female*								
Male	1.022 (0.714–1.463)	0.906	/	/	1.498 (0.914–2.456)	0.109	/	/
Tumor Site								
Left Lung*								
Right Lung	1.293 (0.900–1.858)	0.164	/	/	0.971 (0.590–1.596)	0.906	/	/
Age, years								
≤ 60*								
> 60	1.100(0.771–1.571)	0.599	/	/	1.387(0.837–2.297)	0.204	/	/
Smoking History								
Never-smoker*								
Smokers	0.868 (0.582–1.294)	0.488	/	/	1.190 (0.700–2.024)	0.520	/	/
Tumor Differentiation	2.142 (1.668–2.750)	**0.000**	1.763 (1.361–2.283)	**0.000**	4.041 (2.536–6.438)	**0.000**	2.509(1.475–4.270)	**0.001**
Tumor Size								
≤ 3 cm*								
> 3 cm	0.837(0.584–1.198)	0.331	/	/	0.952 (0.579–1.566)	0.847	/	/
T Stage								
T1-2*								
T3-4	0.924(0.599–1.426)	0.722	/	/	1.098 (0.615–1.962)	0.751	/	/
N Stage								
N0*								
N +	2.949 (2.045–4.251)	**0.000**	2.054 (1.247–3.382)	**0.005**	3.683 (2.148–6.317)	**0.000**	1.210 (0.572–2.562)	0.618
TNM Stage	1.704 (1.358–2.138)	**0.000**	1.202 (0.874–1.655)	0.258	1.896 (1.360–2.642)	**0.000**	1.178 (0.729–1.903)	0.504
Intratumoral MCD								
LOW*								
HIGH	0.937 (0.651–1.349)	0.727	/	/	0.651 (0.380–1.115)	0.118	/	/
MCD Infiltration Ratios								
LOW*								
HIGH	0.435 (0.302–0.625)	**0.000**	0.469 (0.324–0.678)	**0.000**	0.159 (0.085–0.299)	**0.000**	0.241 (0.127–0.457)	**0.000**
Progressive Disease								
NO*								
YES	/	/	/	/	24.840(7.780–79.310)	**0.000**	13.154 (4.015–43.102)	**0.000**

CI: confidence interval; DFS: disease-free survival; HR, hazard ratio; MCD: mast cell density; OS: overall survival

* As control group; Significant *P* values are presented in bold

## Discussion

Because tumors have mast cell infiltration, the connection between mast cells and cancers has received considerable attention. In this study, we evaluated mast cell and CD34 microvessel densities in tumor samples from 238 patients with stage I–III LUAD following complete surgical resection, using an immunohistochemical approach. We concentrated on the two protagonists, LUAD and chymase-positive mast cells, as they had not been the subject of previous studies.

Mast cells have been identified in LUAD tumor tissues, with MCD being used as a discerning metric for mast cell infiltration. The fact that mast cells may promote tumor angiogenesis is supported by the positive correlation between MCD and CD34-MVD, which aligns with findings in other studies on mast cells and angiogenesis in lung cancer [10, 12–14, 25]. Angiotensin I can be activated and converted to angiotensin II, which in turn has the capacity to activate vascular growth factor-1 in malignant tumors by chymase. Additionally, chymase can break down matrix metalloproteinase-9 and indirectly promote angiogenesis [23, 26]. Nevertheless, lung cancer tumor tissues possess both differentiated (CD34 +) and undifferentiated (CD31 + /CD34-) microvessels [27, 28]. Furthermore, MVD, as indicated by CD34, is linked to necrosis and hypoxia [29], suggesting that CD34 is a more suitable marker for mature microvessels. Our results indicated that mature microvessels control mast cell infiltration into tumors via hypoxia in LUAD.

Mast cell chymase is more destructive than tryptase and induces the activation of matrix metalloproteinases, which in turn stimulates the breakdown of the extracellular matrix and aids in tumor invasion and metastasis [21, 23]. This biological action of chymase also adequately explains one of the results of our study: intratumoral MCD was higher in the tumor size ≤ 3 cm group than in the > 3 cm group. In the early stages of tumor formation, mast cells accumulate in the tumor microenvironment. As the tumor grows, mast cells degranulate and release chymase granules into the extracellular space, decreasing the number of mast cells. Chymase plays a role in extracellular matrix degradation to facilitate its penetration by the tumor cells during invasion [23, 30].

Furthermore, we discovered that sex, smoking history, and pathological subtype of LUAD were associated with the level of intratumoral MCD. Estrogen can stimulate an increase in mast cells in the endometrium and cause endometriosis [31]. The higher amount of mast cell infiltration observed in female patients with LUAD may be related to the higher level of estrogen. Patients with a smoking history had reduced intratumoral MCD, suggesting that smoking may diminish the degree of mast cell infiltration in LUAD tumors, possibly because cigarette smoke promotes mast cell degranulation [32]. Different pathological subtypes of LUAD have different intratumoral MCDs. Compared to patients with acinar- and lepidic-predominant adenocarcinomas, those with solid- and micropapillary-predominant adenocarcinomas had a lower degree of mast cell infiltration. Numerous LUAD pathological subtypes correspond to distinct tumor growth patterns. These findings imply that the degree of mast cell infiltration within the tumor may be influenced by variations in the LUAD tumor microenvironment. This distinction encompasses both the internal environmental aspects of the tumor (MVD, tumor size, growth pattern, etc.) and the external environmental factors of the tumor (hormone levels, smoking, etc.).

The results of previous studies evaluating MCD and lung cancer prognosis have differed. Those who used tryptase to identify intratumoral mast cells in lung cancer revealed that patients with high MCD in LUAD had a shorter OS [10]. Imada et al. [13] also revealed that the prognosis of the high mast cell count group was worse in stage I LUAD but not in lung squamous cell carcinoma. Nagata et al. [12] demonstrated the number of tryptase-positive mast cells did not significantly affect postoperative recurrence or survival rates in small-sized LUAD. However, the group with a high number of chymase-positive mast cells experienced worse outcomes than that with a low number of chymase-positive mast cells. In contrast, some studies have found that the prognosis in patients with NSCLC is unrelated to the level of MCD [14, 15], but only in stage I patients; those with a high chymase-positive MCD in the peritumoral area have a decreased mortality risk [14]. Mast cell counts in tumor islets are higher in long-lived patients with NSCLC than in short-lived patients [17]. However, Welsh et al. [16] did not find a connection between mast cell counts in the tumor stroma and survival; however, they showed that the presence of tryptase-positive mast cells in the tumor islets was an independent indication of a favorable prognosis.

Our study observed no correlation between the level of intratumoral MCD in LUAD and patient prognosis, probably because we only counted chymase-positive MCD and did not count MCD in the tumor islets and stroma separately. However, we found that patients with high intratumoral MCD tended to have a better OS rate than those with low intratumoral MCD. Consequently, the different roles played by mast cells in lung cancer may be related to the mast cell phenotype, mast cell micro-position, tumor type, and tumor stage.

Furthermore, we first assessed the prognosis in each patient using the intratumoral/outside MCD infiltration ratio. The findings indicated that patients with a high infiltration ratio had better DFS and OS and that a high infiltration ratio decreased the risk of tumor progression and death in patients with LUAD following complete resection. In addition to mast cells, Evidence suggests that other immune cells infiltrate at different sites in the tumor microenvironment. For example,the distribution distance of T cells close to tumor cells in esophageal cancer [33], the accumulation of intratumoral T cells in clear cell renal-cell carcinoma [34],the high degree of B cell infiltration around the gastric carcinoma [35],the distribution of T cells, B cells and macrophages in different regions of the tumor in triple-negative breast cancer [36],all of them are related to the therapeutic effect and prognosis of tumors. This novel finding of our study regarding differences in spatial distribution may shed new light on the controversial topic of the relationship between mast cells and the prognosis of cancer patients.

Targeting mast cells or their mediators is a potential strategy in cancer therapy. For example, cromolyn sodium, a mast cell stabilizer, can dramatically reduce the growth of thyroid cancer [37]. Medications that target tryptase function, such as tranilast, namostat mesylate, and gabexate mesylate, have antitumor benefits [38]. Mast cell chymase may be a good option for treating lung cancer because low doses of chymase can stimulate the growth of lung cancer cells, whereas high doses have the opposite effect [39].

Our study has some limitations. First, because this was a retrospective study, recollection bias may have affected the data. Second, the “hot spot” approach we used does not accurately represent the overall state of the tumor because it only counts the MCD and MVD in the regions of the tumor with the highest level of expression of the respective markers. Third, our study utilized a small sample size. If more patients were included to increase the sample size and assess whether intratumoral MCD could be utilized as an indicator of patient survival, the results may have differed statistically. Fourth, our findings offer a starting point for the identification of prognostic markers in patients in clinical practice. Multi-center studies with larger sample sizes are required if more promotion is required, and procedures such as counting, interpreting, and slide-reading software should be standardized.

In conclusion, the current study demonstrates that MCD is a quantitative measure of mast cell infiltration, and that intratumoral mast cell infiltration is significantly influenced by variations in the tumor microenvironment of LUADs. Patient prognosis was improved with increasing intratumoral mast cell infiltration levels. In patients with LUAD, the intratumoral or external MCD infiltration ratio can be used as an independent risk factor for postoperative recurrence and death.

## Data Availability

The datasets generated during and/or analysed during the current study are not publicly available due to some reasons but are available from the corresponding author on reasonable request.
